# Construction and validation of a predictive model for the efficacy of valproic acid monotherapy in epilepsy based on Lasso-logistic regression

**DOI:** 10.1016/j.clinsp.2025.100684

**Published:** 2025-07-10

**Authors:** Qichang Xing, Zheng Liu, Haibo Lei, Renzhu Liu, Xiang Liu, Jia Chen

**Affiliations:** a Clinical Pharmacy Department, Xiangtan Central Hospital (The Affiliated Hospital of Hunan University), Xiangtan, PR China; b Zhou Honghao Research Institute Xiangtan, Xiangtan, PR China

**Keywords:** Valproic Acid (VPA), Epilepsy, Efficacy Biomarkers, SNORD3A, Lasso-Logistic Regression

## Abstract

**Introduction:**

Valproic acid (VPA) is a broad-spectrum antiepileptic drug; but its therapeutic efficacy varies significantly among individuals. The objective of this study is to identify the specific biomarkers that can predict the efficacy of VPA.

**Methods:**

The GSE143272 dataset from the Gene Expression Omnibus (GEO) was utilized to identify Differentially Expressed Genes (DEGs) between responders and non-responders to VPA. Weighted Gene Co-expression Network Analysis (WGCNA) was employed to identify genes related to the non-responder phenotype. Intersection genes were selected to obtain the core genes affecting VPA tolerance. Lasso regression was applied to determine the core genes that influence the VPA effect. Lasso regression was applied to screen these core genes, using their expression values as independent variables and VPA response as the dependent variable in constructing a univariate logistic regression model. Peripheral blood samples from epileptic patients treated solely with VPA were collected according to nano-discharge standard. The expression levels of target genes were determined by qPCR to validate the accuracy of the model.

**Results:**

86 genes were closely related to the response phenotype through WGCNA. 13 intersection genes were obtained by intersection with 97 DEGs, which mainly involve mRNA splicing function and transport pathway. Ultimately, 3 genes-NELL2, SNORD3A and mir-1974 were included in the final model. The Area Under Curve (AUC) for this predictive model was found to be 0.70 (95 % CI: 0.70). qPCR analysis revealed a significant difference in the relative expression of the SNORD3A gene between the responder and non-responder groups.

**Conclusion:**

Epilepsy patients are at an increased risk of developing drug resistance when undergoing VPA monotherapy. The risk prediction model based on Lasso-Logistic regression demonstrates strong predictive capabilities. The SNORD3A gene may serve as a valuable biomarker for predicting the likelihood of VPA resistance.

## Highlights

• valproic acid (Depakene) efficacy varies; a predictive model was developed.
• 86 genes linked to VPA response: 3 genes (NELL2, SNORD3A, mir-1974) in final model.
• AUC of 0.70 indicates good predictive ability for VPA resistance.
• qPCR validation confirms SNORD3A as a potential biomarker for VPA efficacy.

## Introduction

Epilepsy is one of the most prevalent chronic neurological disorders, characterized by dysfunction of the central nervous system due to persistent abnormal neuronal discharges.^1^ This condition affects people regardless of age and impacts approximately 50 million individuals worldwide. The recurrent episodes not only harm the health of the patients but also impose significant burdens on their and their families' daily lives, psychological well-being, and financial status. The administration of antiepileptic drugs represents the fundamental and the most effective approach for controlling epileptic seizures; however, about 30 % of patients experience relapse despite adequate treatment.^2^ Valproic Acid (VPA), a non-selective sodium channel blocker, is recognized as the first-line medication for epilepsy management. It has demonstrated favorable outcomes in controlling both generalized and focal seizures; nevertheless, its efficacy is significantly influenced by genetic variations.^3^ The latest research shows that^4^ 100 % of patients with VPA resistance were diagnosed as drug-resistant epilepsy in the late stage, indicating that VPA resistance is an important factor for poor prognosis. Therefore, it is essential to investigate the mechanisms underlying the differential efficacy of VPA in treating epilepsy.

With the advent of the post-genomic era, a new generation of high-throughput sequencing technologies in transcriptomics-such as gene chips and RNA Sequencing (RNA-Seq)-has been widely adopted in medical research, yielding significant advancements.^5^ Bioinformatics methods and Lasso-logistic models have been employed to predict seizures in pregnant women^6^ and drug-resistant epilepsy.^7^

To further investigate the differential effects of VPA on epilepsy treatment from a genetic perspective, this study utilized a dataset from the NCBI Gene Expression Omnibus (GEO), comprising samples from both epilepsy patients and healthy individuals. The authors analyzed and compared transcriptome expression differences in peripheral blood between VPA-responsive and non-responsive patients using bioinformatics approaches to identify Differential Express Genes (DEGs) that may influence VPA efficacy. Subsequently, the authors constructed a VPA response model through Lasso-logistic regression. Peripheral blood samples were collected from 25 epilepsy patients undergoing VPA monotherapy in clinical settings to validate the accuracy of the model, thereby providing a foundation for evaluating VPA efficacy and guiding medication strategies.

## Material and methods

### Data sources

In this study, the dataset with serial number GSE143272 from the GEO database was selected, and the commercial ion channel chip platform GPL10558 was used. The dataset comprised peripheral blood samples from 16 epileptic patients who exhibited a favorable response to VPA monotherapy, 9 epileptic patients who did not respond to VPA monotherapy and 50 healthy controls. Total RNA was extracted from these peripheral blood samples, followed by an analysis of gene expression profiles using the Illumina HumanHT-12 V4.0 expression bead chip.

### Pretreatment and DEGs screening

Based on the information derived from the GPL10558 platform, the probe identification numbers were converted to their corresponding official gene names (retaining only mRNA probes and discarding all non-mRNA probes). The gene expression matrix was analyzed using the limma software package, and DEGs were identified through further processing with Affy and affyPLM software packages. The Benjamini-Hochberg method was employed to control the false discovery rate. A significance threshold of *p* ≤ 0.05 and |log_2_FC| > 0.5 was established as cut-off values. Specifically, log_2_FC > 0.5 indicated up-regulated DEGs, while log_2_FC < −0.5 corresponded to down-regulated DEGs.

### Weighted gene correlation network analysis (WGCNA)

To filter the essential genes, the authors utilized the good SamplesGenes function from the WGCNA package in R. Following this, the authors performed an analysis of a weighted gene co-expression network using WGCNA to obtain a more precise representation of co-expression relationships. The soft threshold power was determined based on principles governing scale-free networks, and the authors employed the recommended soft threshold for subsequent analyses. A dynamic pruning method was applied to uncover co-expression patterns, resulting in a gene cluster tree that illustrates correlations in gene expression. The authors established a minimum module size of 30 genes and subsequently merged modules with similar expression profiles based on their eigenvalue similarity (0.75). Correlation assessments between phenotypes and each module indicated that higher absolute correlation values approaching 1 signified a stronger association between module genes and specific traits. To identify modules significantly associated with particular samples, heat maps were generated for further investigation.

### GO and KEGG enrichment analysis

The intersection of the most relevant module genes and DEGs of the VPA nonresponse phenotype was analyzed by GO and KEGG enrichment.

### Lasso regression screening variables

Take the above intersection targets as candidate variables. The appropriate regularization parameter λ is selected according to the Bayesian Information Criterion (BIC), and only those variables with non-zero regression coefficients are included in the final model. Lasso's L1 regularization path estimation employs a predictor-corrector method, whereby the regression coefficients of each variable are scaled by a uniform factor and rounded to whole numbers, serving as the risk index for assessing poor efficacy in VPA risk scores.

### Construction and evaluation of univariate logistic regression analysis model

Taking the variables screened by Lasso regression as independent variables and whether VPA responded as a dependent variable, a multivariate Logistic regression analysis model was constructed.

### Patient enrolment

The inclusion criteria for this study were as follows: patients diagnosed with primary epilepsy or an epilepsy syndrome who had undergone treatment with VPA monotherapy for a minimum duration of one year. The exclusion criteria encompassed individuals exhibiting impaired liver or kidney function, as well as those suffering from infectious diseases such as upper respiratory tract infections or urinary tract infections within the preceding three months. The subjects ranged in age from 3 to 80 years, irrespective of gender. Following the acquisition of informed consent, participants were registered and monitored in the Department of Neurology of Xiangtan Central Hospital from October 2023 to October 2024. Diagnosis and treatment were conducted by experienced neurologists. According to the International League Against Epilepsy (ILAE) seizure and epilepsy classification scheme^8^ and the research report of Chitra Rawat et al.,^9^ patients included in this study belong to the epilepsy syndrome group and underwent drug concentration monitoring along with dose adjustments. Eyewitnesses, including family members or friends reported on the patient's seizures and relevant historical context. Patients who achieved complete seizure control alongside normal Electroencephalography (EEG) results were classified as responders to VPA; conversely, those experiencing >3 seizures during the observation period were categorized as non-responders. Informed consent was obtained from all participants; if a participant was a minor, written consent was secured from their parents or guardians. The authors collected peripheral blood samples along with clinical and laboratory data while also gathering information regarding their seizure status over the past year.

### Peripheral blood sample processing

At the end of the follow-up period, collect 2‒3 mL blood samples from each patient via a single venipuncture into an EDTA tube (Hunan Univers Medical Technology Co., Ltd, China). Thoroughly mix the whole blood with 0.9 % sodium chloride solution (Sichuan Kelun Pharmaceutical Co., Ltd., China) in a 1:1 ratio. Carefully layer this mixture onto the surface of the layered liquid along the wall of the tube using a pipette. After centrifugation, collect the “cloud layer” to obtain Peripheral Blood Monocytes (PBMCs). Subsequently, add 1 mL TRIzol Reagent (Changsha Dingguo Biotechnology Co., Ltd, China) to fully lyse the cells and transfer them to a 1.50 mL centrifuge tube. Introduce 200 μL trichloromethane solution and vortex gently. Centrifuge for 15 min at 4 °C, with a radius of 8.60 cm at a speed of 12,000 r/min; then collect the supernatant and transfer it to a new centrifuge tube. Add an equal volume of isopropanol and mix thoroughly before allowing it to stand at room temperature for 10 min. Following this step, repeat centrifugation for another 15 min; discard the supernatant and resuspend 1 mL of an ethanol solution prepared with DEPC-treated water at a concentration of 80 %. Centrifuge again for 5 min at 4 °C with a radius of 8.60 cm at a speed of 7500 r/min; invert to air dry briefly before adding 30 μL distilled RNase-free water to dissolve completely. Finally, store samples at −80 °C for future use.

### qRT-PCR validation

Extract a total RNA quantity ranging from 300 to 500 ng. Convert this RNA into cDNA following the instructions provided in the PrimeScript™ RT reagent Kit (Takara Biomedical Technology (Beijing) Co., Ltd., China). Use a fluorescent quantitative PCR instrument (ViiA7 from ABI, USA) along with Hieff™ qPCR SYBR Green Master Mix (No Rox) (Yeasen Biotechnology (Shanghai) Co., Ltd., China) to assess the relative expression levels of the target gene. The primer sequences are detailed in Table 1. Utilize GAPDH or U6 as reference genes and analyze the relative expression of mRNA using the 2^-ΔΔCt^ method. Statistical analysis of data and generation of box-and-whiskers plots are performed using GraphPad Prism 7.0 software. Comparisons between experimental data from two groups are conducted using an unpaired *t*-test. A difference is considered statistically significant when *p* < 0.05.

**Table 1:** Primer sequence.

**Gene**	**Sequence**
H-GAPDH-F	TGTTGCCATCAATGACCCCTT
H-GAPDH-R	CTCCACGACGTACTCAGCG
H—NELL2-F	ACTGCACATGCCTGAATGGA
H-NELL2-R	ACATACGCAAGAGCCGACTT
H-SNORD3A-F	CGAAAACCACGAGGAAGAGA
H-SNORD3A-R	CACTCCCCAATACGGAGAGA
U6-F	CTCGCTTCGGCAGCACA
U6-R	AACGCTTCACGAATTTGCGT
hsa-mir-1974-F	GTGGTTGTAGTCCGTGC
hsa-mir-1974-RT	GTCGTATCCAGTGCAGGGTCCGAGGTATT
	CGCACTGGATACGACATTCTCG
UR	CCAGTGCAGGGTCCGAGGTA

## Results

### Differentially expressed genes (DEGs)

**Fig. 1:**
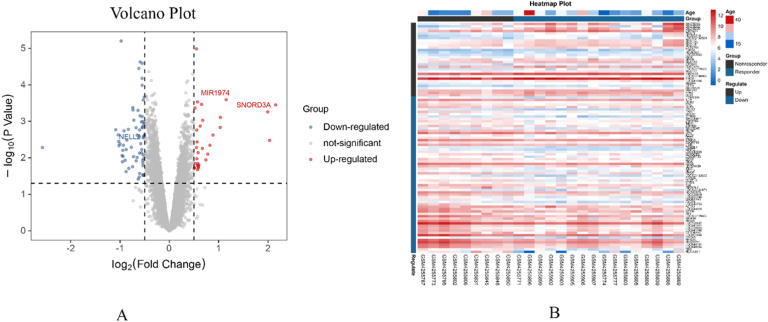
Differential gene analysis volcano map (A) and heat map (B).

Through the screening of DEGs in the GEO dataset GSE143272, DEGs were identified by applying a threshold of p-value ≤ 0.05 and an absolute value of log_2_(FC) greater than 0.5. Among the 97 differential genes, 36 were found to be upregulated while 61 genes were downregulated in the VPA monotherapy response group compared to the non-response group, as shown in Fig. 1.

### Determination of soft threshold of gene co-expression network

**Fig. 2:**
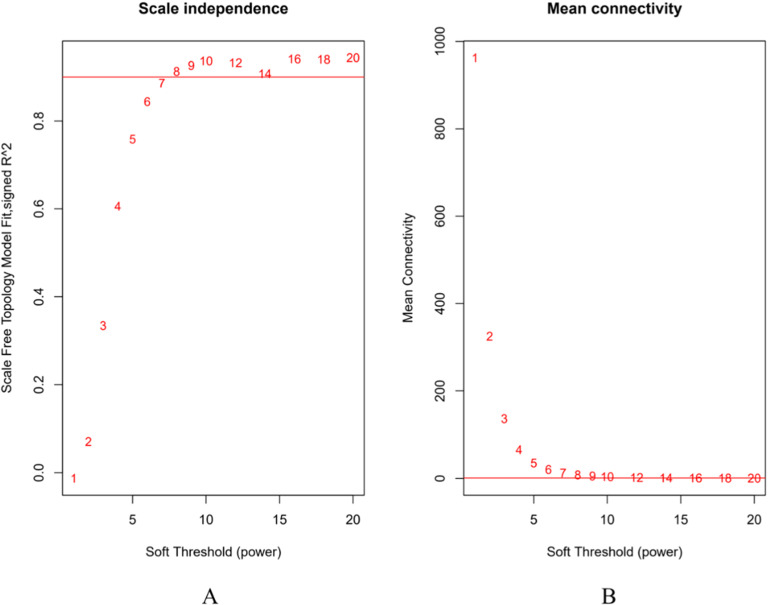
Determination of soft threshold of gene co-expression network. Scale free network fitting index under different soft threshold conditions (A); Network connectivity under different soft thresholds (B).

WGCNA is an important method for in-depth exploration of the functions of key genes amidst data explosion. This technique enables the clustering of genes based on gene expression profile data from various samples, facilitating the identification of co-expressed gene modules across different biological contexts. Subsequently, it allows for the extraction of key genes that exhibit strong correlations with specific traits. To ensure that the co-expression network adheres to a scale-free distribution, the authors employed the function pick Soft Threshold from the WGCNA package in *R* to compute weight values and establish an appropriate soft threshold. The results indicate that the optimal soft threshold β determined by the software is 7, as shown in Fig. 2.

### Gene clustering and module cutting of the gene co-expression network

**Fig. 3:**
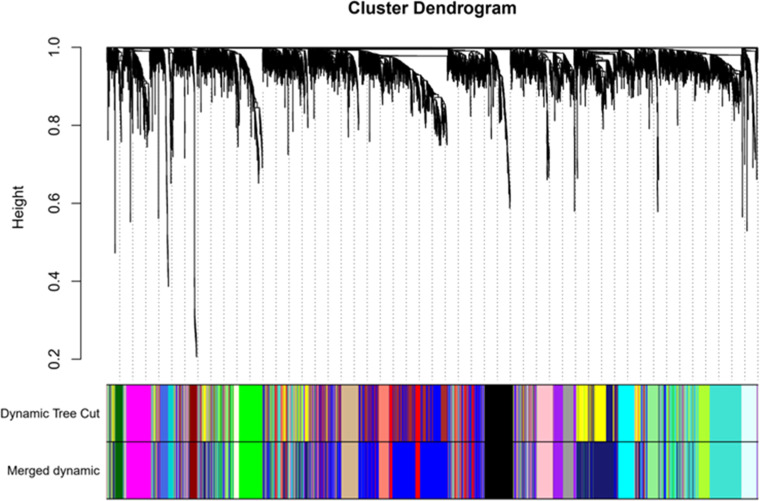
Gene clustering and module cutting of gene co-expression network.

Once the soft threshold β is set to 7, the similarity matrix is converted into a proximity matrix. This proximity matrix is then transformed into a Topological Overlap Matrix (TOM). To accurately change the topology matrix into a dissimilarity matrix, the authors apply the formula dissTom = 1-TOM. This step aids in mitigating errors arising from background noise and spurious correlations. Ultimately, dynamic cutting methods are employed to segment the clusters produced by the hcluter function. Genes exhibiting similar expression patterns are grouped on the same branch representing a distinct co-expression module identified by different colors. Genes that do not conform to any specific module are marked in gray. DEGs are clustered through correlation analysis based on their expression levels, with high-correlation genes assigned to the same module, as shown in Fig. 3.

### Association analysis between gene co-expression network aggregation module and phenotype

**Fig. 4:**
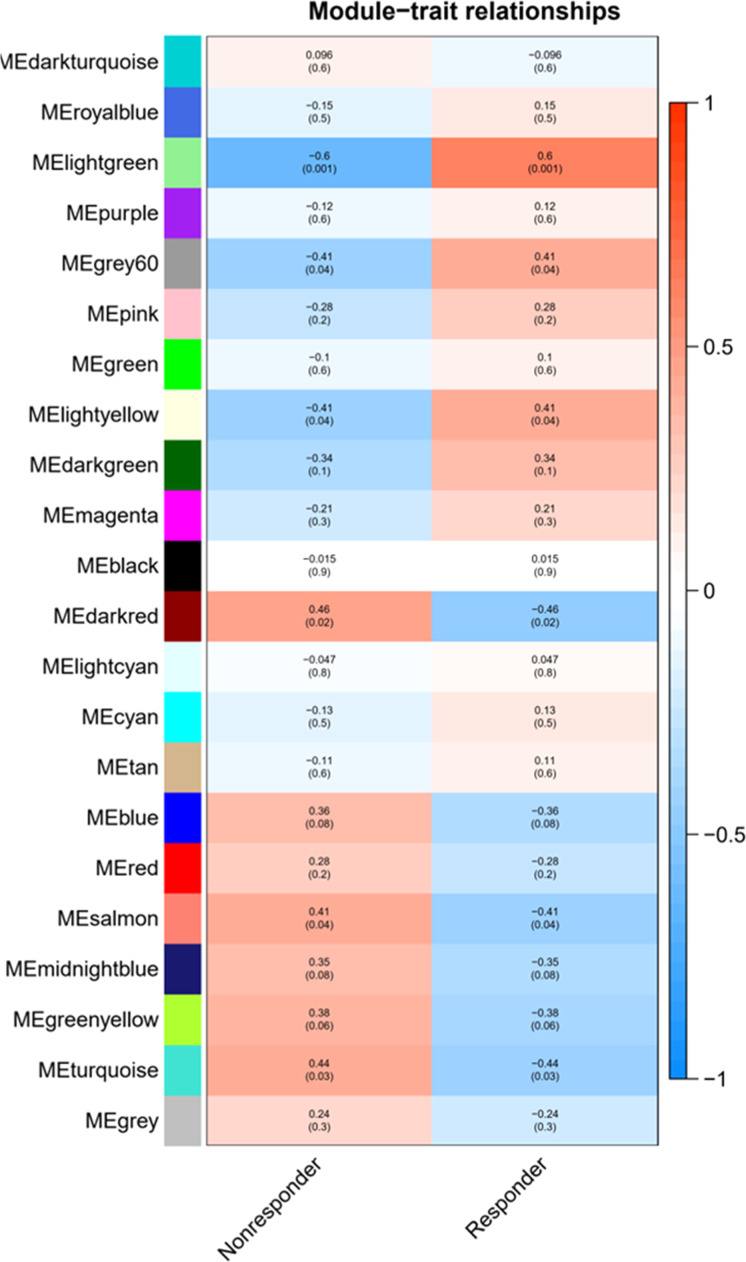
Correlation heat map of gene co-expression network module and different treatments.

**Fig. 5:**
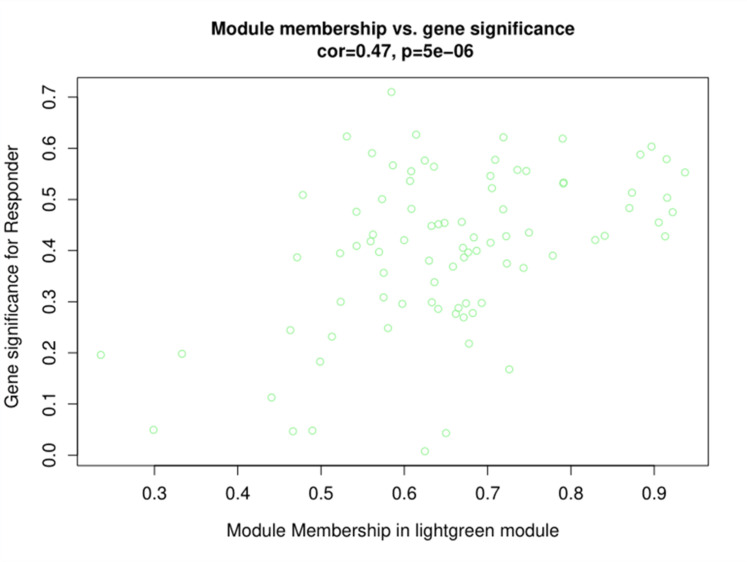
Correlation between light-green module and responder.

After the different treatments are transformed into continuous characters as mass shapes, the correlation analysis is carried out with the module and the correlation heat map is drawn, as shown in Fig. 4. The light-green module exhibits a strong correlation with the VPA response (As shown in Fig. 5), which can be used as the target module for subsequent analysis.

### GO and KEGG enrichment analysis of WGCNA and DEGs intersection genes

**Fig. 6:**
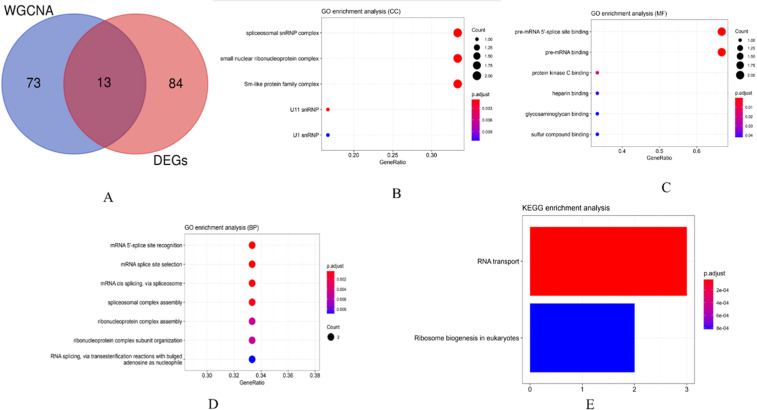
GO and KEGG enrichment analysis of intersection genes.

The intersection genes identified from WGCNA and DEGs were extracted for further analysis. The R package clusterProfiler was used to perform GO enrichment analysis and KEGG enrichment analysis on these intersection genes. The results are shown in Fig. 6. The GO term enriched by the intersection genes predominantly pertains to mRNA splicing sites, while KEGG pathways are primarily associated with RNA transport and ribosomal biogenesis.

### Lasso regression for screening predictive variables

**Fig. 7:**
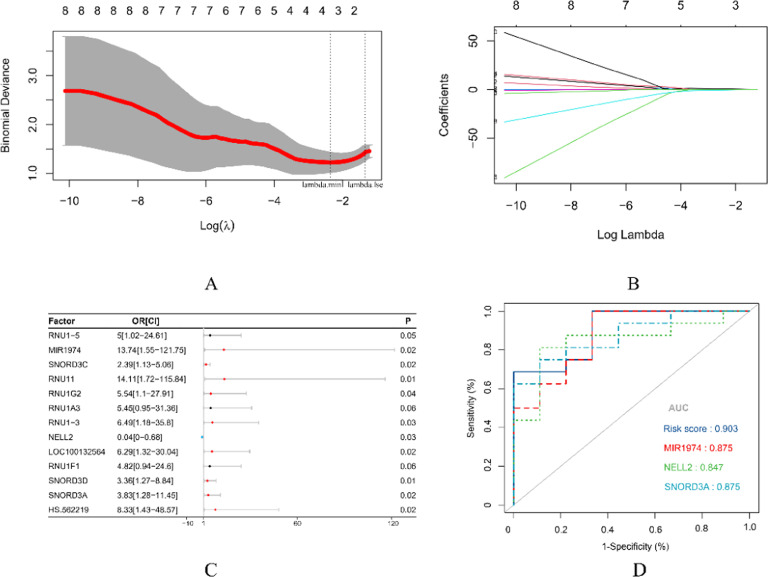
Selection of characteristic variables based on Lasso-logistic regression (A: 10-fold cross-validation diagram; B: Contraction coefficient diagram; C: Univarie logistic regression analysis model; D: ROC curve of risk prediction model).

13 intersection genes were included in the Lasso regression. Fig. 7(A) illustrates the process of determining the optimal λ value through 10-fold cross-validation. In this graph, the vertical axis represents the model's AUC, while the lower horizontal axis displays log(λ), and the upper horizontal axis indicates the number of variables associated with various log(λ) values. The two dashed lines denote specific λ values: lambda.min and lambda.lse. Lambda.min is defined as the λ value that yields the highest AUC, whereas lambda.lse corresponds to a simpler model that maintains an acceptable variance range for AUC reduction. For this study, the authors selected lambda.min = 0.0752 to retain as many variables as possible for further verification. At this time, the variables incorporated into the model are mir-1974, NELL2 and SNORD3A. Each curve in Fig. 7(B) depicts the trajectory of change for each candidate gene. As λ increases, model compression intensifies, resulting in a reduced number of candidate variables entering the model.

### Construction of univariate logistic regression analysis model

The expression levels of 13 candidate genes included in this study across 25 samples were designated as the independent variable, while the response to VPA was utilized as the dependent variable for constructing a single-factor logistic regression analysis model. The results are shown in Fig. 7(C).

### Evaluation of risk prediction model

The Area Under the Curve (AUC) and the 95 % Confidence Interval (95 % CI) of the prediction model were found to be 0.70. The optimal classification threshold for this model is set at 0.54; thus, patients with a predicted expression value greater than 0.54 are classified as responders to VPA treatment. The results are shown in Fig. 7(D).

### qPCR experimental verification

**Fig. 8:**
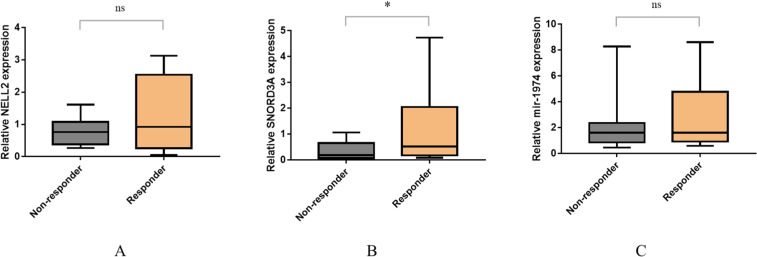
Relative expression of NELL2 (A), SNORD3A (B) and mir-1974 (C) in Non-responders and Responders (* *p* < 0.05, ns: not significant, non-responders compared to Responders).

According to the method of “Patient enrolment”, a total of 25 patients with epilepsy were recruited, comprising 12 responders and 13 non-responders. The relative expression levels of NELL2, SNORD3A and mir-1974 genes were assessed by qPCR and subjected to statistical analysis. The results are shown in Fig. 8. Notably, the relative expression levels of the SNORD3A gene in non-responders were significantly lower than those in responders (*p* < 0.05), which had the same trend as the dataset in the GEO database.

## Discussion

Generally speaking, epilepsy primarily involves dysfunction of ion channels, neuronal damage, inflammatory responses, synaptic plasticity, glial cell proliferation, mossy fiber sprouting, and other neuropathological processes. These factors collectively impact the functionality of neurons in the brain.^10^ An increasing number of epilepsy patients with potential genetic factors have provided substantial evidence suggesting that gene regulation may play a significant role in the pathophysiological mechanisms underlying the onset or progression of epilepsy.^11^, ^12^, ^13^, ^14^

The efficacy of VPA is mainly affected by various clinical and molecular factors, including gender, age, pathogenesis, liver and kidney function, and drug interaction.^15^^,^^16^ In addition to these clinical variables, there are notable differences in the response to VPA among patients. Previous studies have demonstrated that VPA exerts its antiepileptic effect mainly through the three mechanisms: a) Increasing the concentration of inhibitory neurotransmitter Gamma-Aminobutyric (GABA) in the brain to reduce neuronal excitability and control seizures; b) Blocking ion channels and inhibiting ion influx to diminish neuronal excitability; c) Decrease neuronal excitability mediated by N-Methyl-D-Aspartate (NMDA) receptors to mitigate seizures. Any genetic variation affecting these pathways may lead to differences in the therapeutic efficacy of VPA.^17^, ^18^, ^19^ The results predicted in this study appear to diverge from those of previous studies. The analysis utilizing GO and KEGG revealed that the functions associated with WGCNA intersecting with DEGs were predominantly related to mRNA splicing sites. Furthermore, the signaling pathways were chiefly enriched in RNA transport and ribosomal biogenesis processes. This observation may be attributed to epigenetic regulation influencing both susceptibility status epilepticus as well as its severity. Conversely, status epilepticus itself may induce alterations in epigenetic markers that affect gene expression related to epilepsy.

NELL2 is a newly identified gene that encodes six Epidermal Growth Factor (EGF) ‒ like repeats. It was first isolated from a human fetal brain cDNA library by Watanabe et al.^20^ and named due to its similarity to the Neil gene expressed in the nervous system of chickens. NELL2 functions as a secretory protein that is specific to neurons, with predominant expression in the nervous system, particularly within the hypothalamus, where its expression exhibits high selectivity.^21^ Initial research has demonstrated that NELL2 plays a crucial role in facilitating the differentiation and survival of neural cells during embryonic development through the Mitogen-Activated Protein Kinase (MAPK) pathway. Additionally, it promotes the maturation of neural progenitor cells into fully developed neurons.^22^ Abnormal expression levels of NELL2 have been associated with atypical development of hypothalamic neuronal development may lead to irregular excitation patterns among neurons, alterations in neural circuits, and dyssynchrony, ultimately affecting normal nervous system function and increasing susceptibility to epilepsy.^23^, ^24^, ^25^

Mir-1974 is a microRNA (miRNA) found in the human genome, measuring approximately 22 nucleotides in length and capable of regulating gene expression. Its specific functions remain largely unknown. Recent studies have indicated that miRNAs are enriched in mitochondrial RNA and are believed to target numerous tRNA, rRNA and mtDNA coding genes^26^; however, the significance of this phenomenon has yet to be elucidated. Changes in the levels of various miRNAs (such as serum miRNA-4521), have been observed in the hippocampus of individuals with temporal lobe epilepsy as well as within neural tissues of animal models undergoing status epilepticus.^27^, ^28^, ^29^ Following seizures, changes were also noted in the levels of certain miRNAs-including those related to transcription factors and neurotransmitter signaling components in rodent blood samples. In summary, while further research is warranted, mir-1974 may play a crucial role in both the development and management of epilepsy.

LncRNA SNORD3A is a type of nucleolar small RNA (snoRNA) that is involved in the processing of ribosomal RNA precursors. It plays an important role in the onset and progression of viral diseases^30^; however, its involvement in epilepsy remains underexplored in the literature. SNORD3A is classified as a non-coding RNA transcript belonging to the box C/D snoRNA family.^31^ Currently, several functions have been attributed to snoRNA transcripts,^32^ particularly their roles as regulators of stress responses. Research indicates that snoRNA transcripts participate in the formation of protein complexes and contribute to oxidative, metabolic processes, or Endoplasmic Reticulum (ER) stress.^33^^,^^34^ Cellular stress can compromise ER function, which serves as the primary organelle for protein quality control, resulting in an accumulation of misfolded proteins and triggering the Unfolded Protein Response (UPR).^35^ This process represents a coordinated signaling network aimed at restoring ER function when possible while initiating apoptosis if recovery fails. It is widely acknowledged that ER response pathways, including UPR, are crucial within the context of neurodegenerative diseases.^36^ The potential abnormal expression of SNORD3A may reflect irregularities in RNA processing and splicing within neurons, potentially impacting VPA efficacy subject warranting further investigation. Experimental findings indicate that SNORD3A expression levels are significantly lower in peripheral blood samples from VPA non-responders. This suggests that measuring SNORD3A gene expression could serve as a predictive marker for VPA resistance.

Of course, the present research also has some limitations. First of all, since the definition of responder and non-responder treated with VPA is not clear at present, the grouping of patients with epilepsy syndrome treated with VPA monotherapy according to ILAE seizure and epilepsy classification scheme and the research report of Chitra Rawat et al. may not be accurate enough; Secondly, due to the inviolability of brain tissue, this study selected peripheral blood as the research object, which may not well reflect the pathogenesis and drug treatment characteristics of epilepsy; Finally, in view of the fact that there are more choices for the treatment of epilepsy in the current clinical practice, and the cases treated with VPA monotherapy are rare, 25 samples were finally included in this study, which was verified only for the above bioinformatics analysis and prediction model. In the future, the authors will further study the causal relationship, and further verify the current findings by using multi-center and large sample studies.

## Conclusion

In conclusion, patients with epilepsy are at risk of developing drug resistance when undergoing VPA monotherapy. The risk prediction model based on Lasso-logistic regression demonstrates strong predictive capability. Furthermore, the SNORD3A gene may serve as a valuable biomarker for predicting VPA resistance, thereby facilitating targeted combination therapies or adjustments in medication to achieve precise outcomes.

## Ethics approval and consent to participate

This experiment was approved by the ethics committee of Xiangtan Central Hospital (approval No. 2023–09–001). This study is an Observational Study and follows the STROBE Statement.

## Authors’ contributions

Xing Qichang, Liu Zheng: Conceptualization; methodology; software.

Chen Jia: Data curation; writing-original draft preparation.

Lei Haibo: Visualization; investigation.

Liu Xiang: Supervision.

Liu Renzhu: Writing-reviewing and editing.

## Funding

This work was supported by the scientific research program of Hunan Provincial Health Commission (Grant No. B202313018450).

## Declaration of competing interest

The authors declare no conflicts of interest.
